# The *OsGAPC3* mutation significantly affects grain quality traits and improves the nutritional quality of rice

**DOI:** 10.3389/fpls.2024.1470316

**Published:** 2024-10-03

**Authors:** Bo Peng, Yan Liu, Xiaoyu Sun, Qiang Zhao, Jing Qiu, Xiayu Tian, Jing Peng, Zhiguo Zhang, Yujian Wang, Yaqin Huang, Ruihua Pang, Wei Zhou, Yuliang Qi, Yanfang Sun, Quanxiu Wang, Yuqing He

**Affiliations:** ^1^ College of Life Sciences, Xinyang Normal University, Xinyang, China; ^2^ Henan Scientific Research Platform Service Center, Zhengzhou, China; ^3^ College of Agronomy, Xinyang Agriculture and Forestry University, Xinyang, China; ^4^ Henan Lingrui Pharmaceutical Company Limited, Xinyang, China; ^5^ School of Pharmacy, Xinyang Agriculture and Forestry University, Xinyang, China; ^6^ Xinyang Academy of Agricultural Science, Xinyang, China; ^7^ National Key Laboratory of Crop Genetic Improvement, Huazhong Agricultural University, Wuhan, China; ^8^ Hubei Hongshan Laboratory, Wuhan, China

**Keywords:** *OsGAPC3*, rice, nutritional quality, grain quality, salt-stress response

## Abstract

The glycolytic enzyme cytoplasmic glyceraldehyde-3-phosphate dehydrogenase (GAPC3) is involved in multiple biological processes in plants, including transcriptional regulation, and material metabolism. However, the relationship between *OsGAPC3* and the quality traits of rice is poorly understood. Here we identify *OsGAPC3* mutations that enhance the protein content and grain nutritional quality of rice by regulating the *OsAAP6* gene expression. The number and volume of type-II protein bodies in the endosperm of the *OsGAPC3* mutants, and GPC increase significantly. We report significant increases in chalkiness area and degree, and decreases for starch content, gel consistency, and taste value. Results of proteomic detection and analysis reveal that *OsGAPC3* affects the major storage substances (proteins and starch) metabolism in rice, and the accumulation of proteins and starch in the endosperm. Additionally, the *OsGAPC3* mutation significantly decreases the rice-seedling salt tolerance. Therefore, *OsGAPC3* affects multiple quality traits of rice, participates in regulating rice-seedling salt-stress response. These data can be used to design better-quality and stronger salt-resistant rice varieties.

## 1 Introduction

Rice (*Oryza sativa* L.) is a globally important food crop, with more than half of the world’s population dependent on it as a staple food (Li P. et al., 2022). In China, over 60% of the population is dependent on rice as a dietary staple (Wing et al., 2018). While significant progress has been in improving rice yield (Zhou et al., 2019), a more-discerning population also demands improved the grain quality of rice.

Rice grain quality refers to various grain characteristics affected by production, processing, sale, and consumer cooking and consumption, such as grain nutritional, appearance, processing, cooking, and eating qualities (Li et al., 2018; Custodio et al., 2019; Zhao et al., 2022; Li Y. et al., 2022). The quality of appearance is largely affected by the grain shape, and endosperm opacity (or chalkiness). Rice-grain type is a typical quantitative trait that is controlled by various genes, and it is affected by the environment (Zhao et al., 2022). Processing quality mainly indicates the state of rice after milling and processing, and is principally determined by milled rice, brown rice and head rice rates (Peng et al., 2024a). The brown rice rate indirectly indicates a grain’s edible quality, and head rice rate is a key indicator of rice grain quality (Tong et al., 2019; Peng et al., 2023). The cooking and eating quality of rice refers to the physicochemical properties during the cooking process, such as swelling, water absorption, retrogradation, color (luster and whiteness), gelatinization, morphology (grain structure and integrity), aroma, and palatability (viscosity, elasticity, and softness) (Zhou et al., 2019). Physicochemical indicators for measuring the taste quality of cooking include amylose content (AC), gel consistency (GC), and gelatinization temperature (GT) (Zhou et al., 2019; Bao et al., 2023). Nutritional quality mainly depends on nutrient levels such as protein, starch, fat, amino acids, vitamins, and trace elements. The digestible protein in rice accounts for over 99% of the total protein content, and it is a high-quality protein that is easy for the human body to digest and absorb. Its composition and content are important indicators of the grain nutritional quality of rice (Riaz et al., 2018; Peng et al., 2021; Jayaprakash et al., 2022). Grain protein is the second largest component in rice seeds, and its content is a crucial factor in determining a rice’s nutritional quality. According to their function, proteins in the endosperm can be divided into (mostly) storage and (secondarily) structural proteins. Depending on the solubility of storage proteins, they can be divided into globulin, albumin, prolamin, and glutenin (He et al., 2013; Chen et al., 2018). Storage proteins are the main source of nutrients for rice seed germination and seedling growth.

Amino acid transporter (AAT) plays a critical role in the transport of plant amino acids. Amino acid permease (AAP) is a key member of the AAT family, and AAPs play an important role in amino acid transport processes in different plant organs (Tegeder et al., 2017; Peng et al., 2024b). To date, 19 *OsAAPs* have been identified in rice. Of these, *OsAAP1* and *OsAAP4* play important roles in regulating the redistribution of multiple amino acids, facilitating amino-acid transport to reproductive organs, maintaining spikelet fertility, and regulating growth and rice yield (Ji et al., 2020; Fang et al., 2021; Gomes et al., 2022). *OsAAP3* and *OsAAP5* negatively regulate rice tillering and yield (Lu et al., 2018; Wang et al., 2019; Wei et al., 2021). *OsAAP6* can positively regulate grain protein content (GPC), thereby affecting grain quality (Peng et al., 2014). *OsAAP7* and *OsAAP16* can effectively transport multiple amino acids in rice (Taylor et al., 2015). *OsAAP8* mutation significantly increases rice grain protein, amino acid contents, and nutritional quality (Peng et al., 2024b). *OsAAP10* regulates the starch and protein contents of rice grains by affecting acidic amino-acid transport (Wang et al., 2020). *OsAAP11* mainly affects the content of neutral amino acids in grains, thereby regulating their amino acids and GPC (Yang Y. H. et al., 2023). *OsAAP13* is involved in the transportation of various amino acids in above-ground parts of rice, and their growth and development (Nie et al., 2023). *OsAAP14* and *OsAAP15* have significant impacts on the yield and related traits of rice (Yang et al., 2022; Yang et al., 2023). *OsAAP17* alleles can positively regulate rice yield in the *indica* variety, especially in the dry season (Nayak et al., 2023). Therefore, *OsAAPs* play a critical role in amino-acid transport and redistribution in different organs. They participate in regulating rice growth, development, material metabolism, and play an important role in signal transduction, collectively affecting rice yield and quality.

Glyceraldehyde-3-phosphate dehydrogenase (GAPDH) is a critical enzyme in glycolysis and gluconeogenesis sugar metabolism pathways, and plays a core role in cellular carbon metabolism. Mammalian GAPDH has various non-glycolytic functions such as membrane lysis, phosphotransferase activity, DNA binding and repair, cell apoptosis (Tossounian et al., 2020), nuclear RNA output, microtubule bundling, and in the cell cycle (Kosova et al., 2017; Hyslop and Chaney, 2022). The four GAPDH subtypes in higher plants are chloroplast glyceraldehyde 3-phosphate dehydrogenase (GAPA/B), plastid glyceraldehyde 3-phosphate dehydrogenase (GAPCp), cytoplasmic glyceraldehyde 3-phosphate dehydrogenase (GAPC), and non-phosphorylated glyceraldehyde 3-phosphate dehydrogenase (NP-GAPDH) (Rius et al., 2008). The GAPA/B subtypes mainly participate in processes such as thiol modification and photosynthesis (Fermani et al., 2007). In monocotyledonous graminaceous plants, GAPC plays an important role in plant growth, development, and the stress response (Kim et al., 2020; Sajeesh et al., 2021; Kim et al., 2022). In each of the seven phosphorylated GAPDHs in the Thale cress *Arabidopsis* genome, cytoplasmic *GAPC* is expressed in all plant organs, particularly in the stems, leaves, and pods (Hildebrandt et al., 2015). Additionally, the *GAPC* of *Arabidopsis* is associated with the cytoskeleton and participates in energy metabolism and functional regulation (Giegé et al., 2003). Of three cytoplasmic GAPDH sequences (*OsGAPC1–3*) identified in the rice genome, *OsGAPC2* interacts with ubiquitin ligase EL5 to prevent rice root meristem cell death under high nitrogen conditions. Overexpression of *OsGAPC3* gene can enhance rice salt tolerance (Nishizawa et al., 2015; Zhang X.H. et al., 2011). Nevertheless, it remains unclear if *OsGAPC* gene was involved in rice biological processes such as seed growth and development, as well as material metabolism.

We initially isolated and cloned a major QTL (quantitative trait locus) *OsAAP6* (*qPC1*) that positively regulated grain protein content in rice (Peng et al., 2014). To elucidate the molecular genetic mechanism of the *OsAAP6* gene, we screened and confirmed the interaction between OsGAPC3 and *OsAAC6* using yeast one hybrid, local surface plasmon resonance experiments, and *in vivo* point-to-point experiments. In rice *OsGAPC3* gene edited transgenic plants, we found that *OsGAPC3* affected the content of main storage substances (starch and protein) in rice grains by regulating expression of *OsAAP6*, and significantly affected various grain-quality traits. Moreover, *OsGAPC3* was involved in rice-seedling salt-stress response, this indicated that *OsGAPC3* had various biological functions, which is of importance for molecular breeding of new rice varieties.

## 2 Materials and methods

### 2.1 Point-to-point experiments in yeast

The bait plasmid pHIS2-*OsAAP6* (the promotor region from −377 bp to the transcription start site of the *OsAAP6*) and positive clone plasmid pGADT7-Rec2-*OsGAPC3* were simultaneously transformed into Y187 yeast cells and cultured at 30°C for 4 days. We then selected yeast monoclonal antibodies and inoculated them into liquid culture medium, and cultured them for 12 h. After diluting the cell solution, it was spotted on solid culture medium (150 mmol·L^−1^ 3AT, SD-Leu-Trp-His) and incubated for 4 days (Peng et al., 2023, 2024b). The OsGAPC3 yeast colony thrived on the plate (containing 3AT inhibitors), which was clearly different from the negative control (-) and compatible with the positive control (+).

### 2.2 Local surface plasmon resonance validates that OsGAPC3 can directly bind to the *OsAAP6* gene

A surface plasmon resonance biosensor located using OpenSPR was used for OsGAPC3 and the *OsAAP6* gene binding tests. We fixed the *OsAAP6* gene antibody on the COOH sensor chip and then injected the blocking solution. All of the cell lysate protein was injected into the sensor chip, and the sensor captured the *OsAAP6* gene (Hou et al., 2023). Then, the 50 μg·mL^-1^ IgG (negative control) was injected into the sensor chip, and different concentrations (15.0 μg·mL^-1^, 22.5 μg·mL^-1^, 30.0 μg·mL^-1^) of OsGAPC3 antibody as the target protein to observe the interaction with the *OsAAP6* gene.

### 2.3 Creation of *OsGAPC3* mutant plants

To obtain *OsGAPC3* mutant plants, the CRISPR/Cas9 tool was exploited to target the fourth to fifth exons of the Zhonghua 11 (ZH11) *OsGAPC3* gene, the DNA fragment containing the target sites were amplified by PCR (**Supplementary Table S1**) and cloned to the final CRISPR expression vector PRGEB32-TRNA. The *OsGAPC3* mutant plants were obtained by agrobacterium mediated genetic transformation (Peng et al., 2024b). The Sanger sequencing was used to validate the *OsGAPC3* mutant plants (*Osgapc3-1*, *Osgapc3-2* and *Osgapc3-3*) (**Supplementary Figures S2B, S3**).

### 2.4 Quantitative real-time PCR

qPCR was performed in a total volume of 25 μL containing 2 mL of the reverse-transcribed product, 0.2 mmol·L^−1^ of gene specific primers (**Supplementary Tables S1–S4**), 12.5 μL of SYBR Green Fast qPCR Master Mix, and 0.5 μL of Rox Reference Dye II on an ABI 7300 quantitator (Peng et al., 2023). The rice *β-actin* gene was used as the control, and relative expression levels were standardized using the 2*^−ΔΔCT^* calculation method (Peng et al., 2014). All data obtained by qPCR are based on three biological replicates.

### 2.5 *OsGAPC3* expression analysis

According to the cDNA sequence of *OsGAPC3*, the primer was designed by NCBI software (www.ncbi.nlm.nih.gov/home/tools/). The total RNA was extracted from various rice tissues (including roots, leaves, glumes, stems, eustipes, flag leaves, and endosperm) at different stages with the RNA extraction kit (Invitrogen, China). The first strand cDNA was synthesized in a volume of 10 μL. Protocols for qPCR are as described in section 2.4.

### 2.6 Subcellular localization of the OsGAPC3 protein

The amplified *OsGAPC3* cDNA fragment was connected to the pM999 vector (GFP) to obtain *OsGAPC3*-*GFP*, and PEG-mediated transfections were performed (Zhang H. et al., 2022). Protoplasts were observed using a confocal fluorescence microscopy (ECLIPSE 80i). All fluorescence experiments were independently repeated three times.

### 2.7 Plant growth conditions and trait measurement

Rice plants were examined under field conditions at the experimental stations of Xinyang Normal University (XYNU). Fields were managed according to local (Xinyang, Henan) agricultural practices (Peng et al., 2023). Harvested rice seeds were stored at room temperature for 3 months (Peng et al., 2024a). Then, the rice grain quality traits were measured (Li et al., 2014). Milled rice was ground to flour for measurement of gel consistency (GC), and starch content. The GPC, taste value, free fatty acids, and amylose content (AC) were analyzed by near infrared grain analyzer (Peng et al., 2024b). Data were analyzed using Microsoft Excel software.

### 2.8 Transmission electron microscopy analyses

Transverse sections of rice seeds (10 DAF) were fixed in 2.5% glutaraldehyde buffered for 12 hours, then processed and sectioned using a frozen section technique. The protein bodies (PBs) in endosperm were observed by transmission electron microscopy (Tecnai G2 F20), and the area of protein bodies was determined using ImageJ software.

### 2.9 Scanning electron microscopy observation

The middle of the rice grain was tapped with the back of a knife blade to make a natural break, the broken part of the grain was then cut with a knife to make a sample of 2–3 mm thick (Peng et al., 2024b). One part of the grain was observed by optical microscope, the other part of the grain section was observed by scanning electron microscopy (Hitachi S-4800).

### 2.10 Proteomic analysis

Homozygous mutants of *OsGAPC3* and wild-type rice were cultured under the same growth environment, and endosperms of the same period were taken for iTRAQ-based proteomic sequencing analysis. Principal component analysis and relative standard deviation distributions were performed on all samples after sequencing was completed to determine the reliability of proteome sequencing data. Screen differentially expressed proteins by fold change (FC) of repeated quantitative values and *P*-value corresponding to relative quantitative values. After the localization analysis and COG database annotation of the differentially expressed proteins, GO functional enrichment analysis and KEGG metabolic pathway enrichment analysis were performed, respectively (Liu et al., 2021).

### 2.11 Salt stress test

The *OsGAPC3* mutants and the wild-type seedlings were cultured in the nutrient solution containing different salt concentrations (50 mmol·L^−1^ and 100 mmol·L^−1^) in a 28°C constant-temperature light incubator for 7 days. The nutrient solution was renewed every two days. The growth of *OsGAPC3* mutants and wild-type seedlings was recorded daily (Luo et al., 2023).

## 3 Results

### 3.1 OsGAPC3 can interact with *OsAAP6*

**Figure 1:**
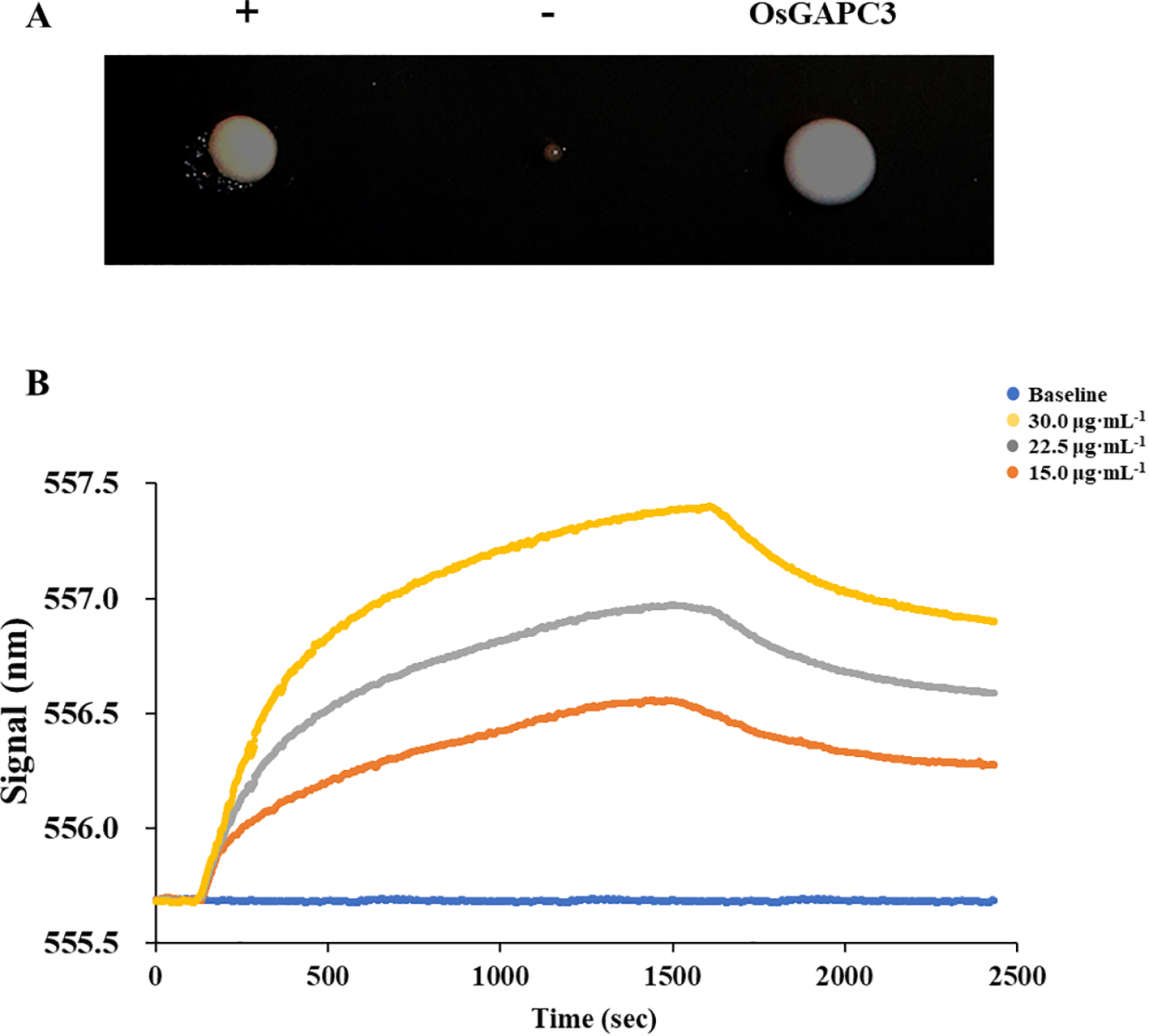
Validation of interactions between *OsAAP6* and OsGAPC3. **(A)** verification of the interaction between OsAAP6 and OsGAPC3 in yeast point-to-point experiments. **(B)** *In vitro* validation of interactions between OsGAPC3 and *OsAAP6* via localized surface plasmon resonance. The affinity values of *OsAAP6* with OsGAPC3 antibody at concentrations of 15.0 μg·mL^-1^, 22.5 μg·mL^-1^, 30.0 μg·mL^-1^ were 4.42×10^-5^ KD, 5.57×10^-6^ KD and 1.41×10^-6^ KD, respectively.

Initially, a total of 92 regulatory factors that may bind to the *OsAAP6* gene were screened by yeast one hybrid system (Peng et al., 2023). The bait plasmid (pHIS2-*OsAAP6*) and the plasmid pGADT7-Rec2-*GAPC3* were simultaneously transferred into the Y187 (yeast cell). The growth of OsGAPC3 yeast colonies with added 3AT inhibitors was good, and differed significantly from the negative control (-) (**Figure 1A**). This indicated that the OsGAPC3 could bind to the promoter region (from −377 bp to the transcription start site) of *OsAAP6* in yeast.

To further test the interaction between OsGAPC3 and the *OsAAP6* gene, the pET-28a and pET-28a-*OsGAPC3* were simultaneously transfected into the competent cells BL21 (*Escherichia coli*). Through local surface plasmon resonance experiments, we determined that the curve significantly trended upward at 150 s, indicating a binding effect between OsGAPC3 and *OsAAP6*, forming a complex; then the curve trended downward at 1500 s, showing that the OsGAPC3-*OsAAP6* complex underwent dissociation (**Figure 1B**). The affinity values of *OsAAP6* with OsGAPC3 antibody at different concentrations (15.0 μg·mL^-1^, 22.5 μg·mL^-1^, 30.0 μg·mL^-1^) were 4.42×10^-5^ KD, 5.57×10^-6^ KD and 1.41×10^-6^ KD, respectively (**Figure 1B**). The above results indicate that OsGAPC3 could interact with *OsAAP6 in vitro*.

### 3.2 *OsGAPC3* is a constitutively expressed gene and OsGAPC3 protein may be localized in the cytoplasm of rice

**Figure 2:**
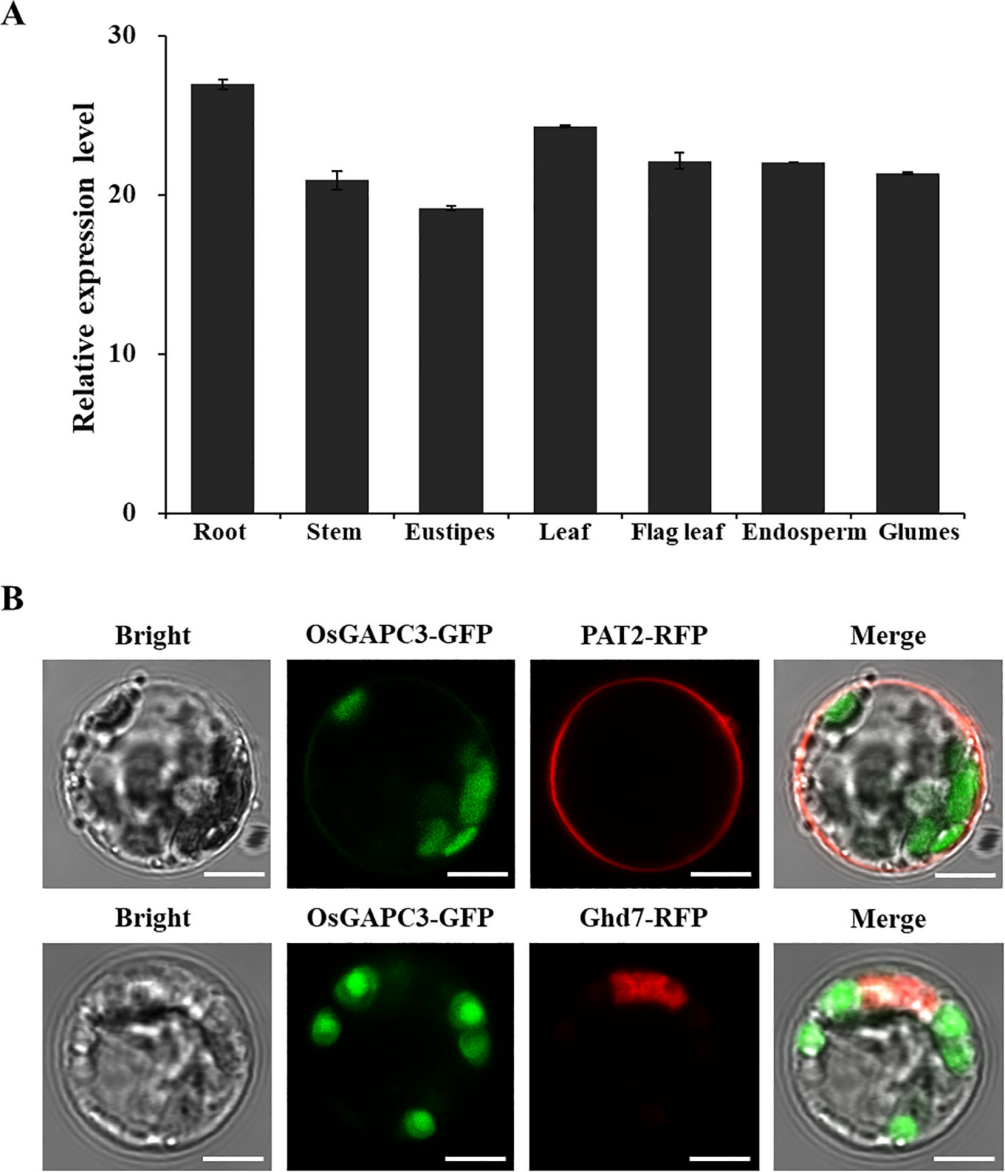
Analysis of *OsGAPC3* expression patterns and subcellular localization in rice. **(A)** Detection and analysis of expression levels in different tissues. Error bars, standard error of the mean. **(B)** OsGAPC3-GFP is not co-localized with membrane marker protein PAT2-RFP or nuclear marker protein Ghd7-RFP. Bars: 5 μm.

To detect the expression pattern of *OsGAPC3* gene in different tissues of rice, RNA was extracted from the rice root, stem, eustipes, leaf, flag leaf, endosperm and glumes. The qPCR results indicate that *OsGAPC3* was expressed in each tissue, which is indicative of a constitutive expression gene. *OsGAPC3* has a higher expression level in roots and glumes, but lower expression levels in stems and eustipes (**Figure 2A**). These qPCR detection results are consistent with the expression level of *OsGAPC3* in the CREP database (http://crep.ncpgr.cn/crep-cgi/home.pl) (**Supplementary Figure S1**).

To detect localization of OsGAPC3 in rice cells, *OsGAPC3* was fused with the green fluorescent protein gene (*OsGAPC3*-*GFP*), and co-expressed with the *PAT2*-*RFP* (cell membrane localization marker) and the *Ghd7*-*RFP* (nuclear marker) in rice protoplasts, then observed under confocal electron microscopy. Because OsGAPC3-GFP did not co-localize with Ghd7-RFP (nuclear marker protein) and PAT2-RFP (membrane marker protein) (**Figure 2B**), OsGAPC3 may be localized in the cytoplasm.

### 3.3 *OsGAPC3* mutation leads to significant changes in rice grain protein and starch content

To elucidate the function of *OsGAPC3* gene in rice, the *OsGAPC3* mutation plants were obtained by CRISPR/Cas9 tool. We first conducted off target prediction through the website (http://crispor.tefor.net/crispor.py) and found that the off target scores of sgRNA were consistently low (LOC_Os10g15180:0.117, LOC_Os06g43420: 0.113, LOC_Os05g05580: 0.111). Subsequently, we conducted sequencing analysis on these three genes that may have off target effects, and found that none of them changed, indicating that no off target effects occurred during the *OsGAPC3* gene editing process. A total of 24 CRISPR/Cas9 gene edited plants were obtained, including 18 *OsGAPC3* mutant plants (**Supplementary Figure S2A**). After DNA sequencing, three types of *OsGAPC3* mutants were identified: *OsGAPC3-1*, *OsGAPC3-2*, and *OsGAPC3-3* (**Supplementary Figures S2B, S3**). The *OsGAPC3-1* has a deletion of 6 bp in its fourth exon, resulting in a deletion mutation; *OsGAPC3-2* has a deletion of 196 bp, resulting in a frameshift mutation; *OsGAPC3-3* has an insertion of 1 bp and a deletion of 1 bp, resulting in a frameshift of the 200 bp sequence between target sequences (**Supplementary Figures S2B, S3**).

**Figure 3:**
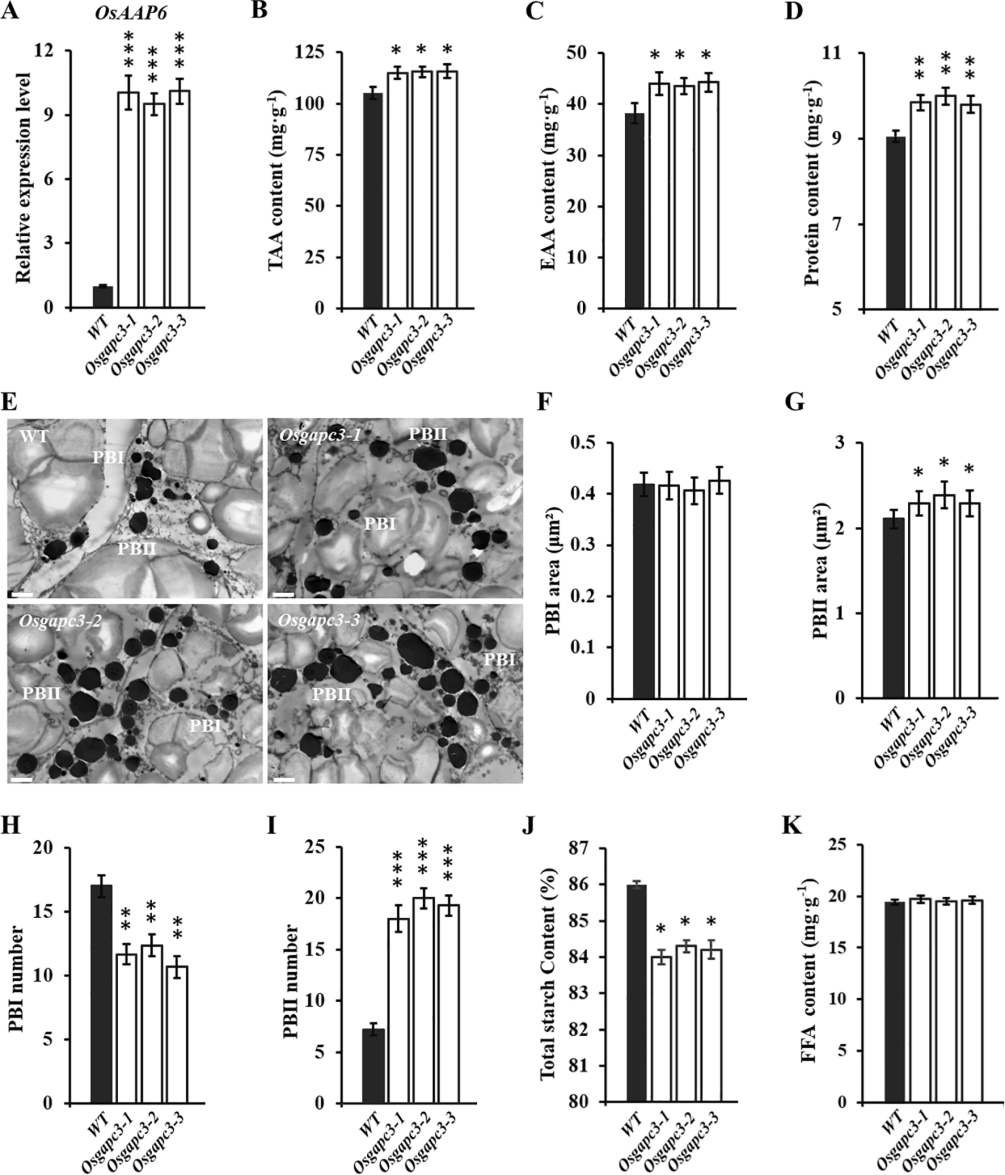
Detection of nutritional quality in grains of *OsGAPC3* mutants. **(A)** Detection and analysis of expression levels of *OsAAP6* in *OsGAPC3* mutants. **(B)** Detection and analysis of total amino acid content in the seeds of *OsGAPC3* mutant. TAA, total amino acid. **(C)** Detection and analysis of essential amino acid content in the seeds of *OsGAPC3* mutant. EAA, essential amino acid. **(D)** Detection of GPC in *OsGAPC3* mutants. **(E)** Transmission electron microscopy observation of endosperm 5 DAF of *OsGAPC3* mutants, scale: 2 μm. Average cross-sectional area of **(F)** PB I and **(G)** PB II. Statistical analysis of the quantity of **(H)** PB I and **(I)** PB II. **(J)** Detection of total starch content in grains of *OsGAPC3* mutants. **(K)** Detection and analysis of free fatty acid content in *OsGAPC3* mutant seeds. Significant differences based on two-tailed *t*-test: ^***^ *P* ≤ 0.001, ^**^ *P* ≤ 0.01, ^*^ *P* ≤ 0.05. WT, Wild type. Bars: 2 μm. Error bars, standard error of the mean, n ≥ 50.

To clarify expression levels of *OsAAP6* in *OsGAPC3* mutants, qPCR detection was performed. Expression levels of the *OsAAP6* gene were significantly increased in *OsGAPC3* homozygous mutants (T_2_) (**Figure 3A**). Because of the influence of the *OsAAP6* gene on the distribution of many amino acids in rice as well as its positive regulation of GPC, we examined protein and amino acid contents in grains of *OsGAPC3* mutants. The total amino acid (TAA) content and essential amino acid (EAA) content of *OsGAPC3* homozygous mutant seeds were significantly increased (**Figures 3B, C**).

The GPC of *OsGAPC3* homozygous mutants increased (**Figure 3D**). To further investigate if *OsGAPC3* mutation affects protein bodies (PB) development in the rice endosperm, we examined the endosperm of *OsGAPC3* mutants 5 DAF using transmission electron microscopy. Both PB I and PB II were clearly visible, with PB I showing relatively regular circular, and PB II being irregular and uniformly stained (**Figure 3E**). There was no difference in cross-sectional area of PB I in the endosperm of *OsGAPC3* homozygous mutants compared with the wild-type, but quantity was significantly reduced. The cross-sectional area and quantity of PB II significantly increased (**Figures 3F–I**). Thus, the increased volume and quantity of PB II in *OsGAPC3* mutants likely contributed to increased GPC and nutritional quality.

To further clarify if *OsGAPC3* affects the accumulation of other nutrients in rice, total starch, and free fatty acid (FAA) in *OsGAPC3* mutant grains were measured. Because total starch content in *OsGAPC3* homozygous mutants was markedly reduced (**Figure 3J**), and there was no change in FAA content (**Figure 3K**), the *OsGAPC3* mutation helped increase GPC, but was not conducive to starch accumulation in grains.

### 3.4 *OsGAPC3* affects the expression of genes related to starch and protein metabolism

Because of the impact of *OsGAPC3* mutations on the accumulation of main storage substances (starch and protein) in grains, the expressions of 20 genes related to starch and protein metabolism in *OsGAPC3* mutants (T_2_) were examined. Expression levels of genes that positively correlate with GPC synthesis metabolism (e.g., *10KD Prolamin*, *AlaAT*, *Globulin1*, *Glutelin 4*, *GluA3*, *GluA1*, *GluB1*, *11S Globulin*, *GluA2*, *GluB5*) were all upregulated in *OsGAPC3* homozygous mutants (**Supplementary Figures S4A–J**). Additionally, expression levels of the starch synthesis related genes (*Susy2* and *Susy3*) were significantly downregulated, while those of the starch degradation metabolism related genes (*ISA1*, *ISA2*, *AMY3A*, *AMY3B*, *SSIIa*) were upregulated (**Supplementary Figures S4K–T**). These results are consistent with the phenotype that includes a significant increase in the grain protein content and a corresponding decrease in starch content of *OsGAPC3* homozygous mutants.

### 3.5 *OsGAPC3* mutation affects rice appearance, cooking, and eating qualities

**Figure 4:**
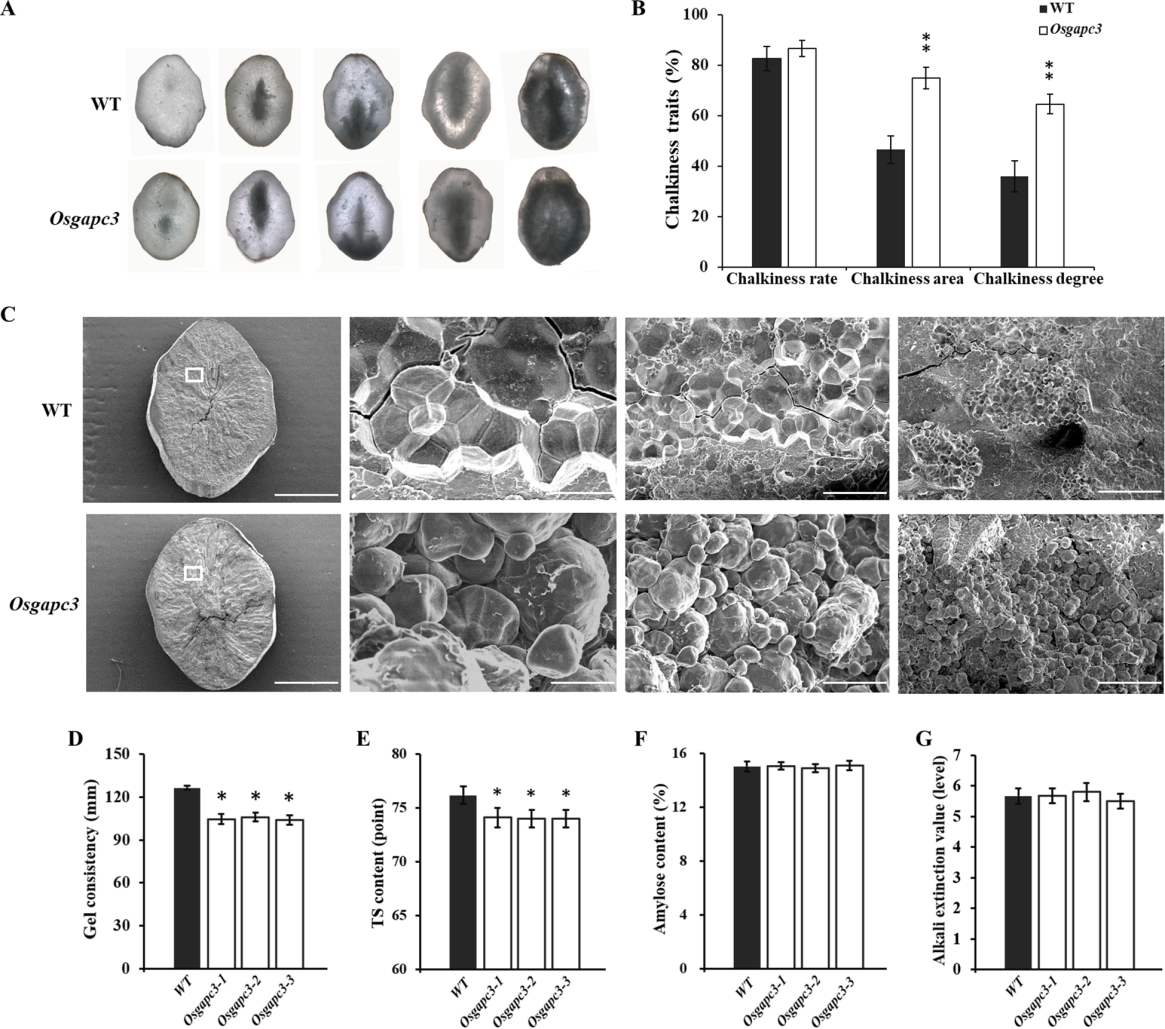
Detection and analysis of chalkiness traits in *OsGAPC3* mutants. **(A)** Endosperm chalkiness under optical microscope (40×). **(B)** Statistical analysis of endosperm chalkiness in *OsGAPC3* mutants. **(C)** Endosperm of *OsGAPC3* mutants under scanning electron microscopy (scales left to right: 1 mm, 10 μm, 20 μm, and 100 μm). **(D)** Rice gel consistency. **(E)** Taste value. **(F)** Amylose content. **(G)** Rice gelatinization temperature. Significant differences based on two-tailed *t*-test: **P ≤ 0.01, *P ≤ 0.05. WT, Wild type. Error bars, standard error of the mean.

Because of the impact of *OsGAPC3* mutations on the accumulation of main storage substances (starch and protein) in grains, we investigated chalkiness traits in *OsGAPC3* mutants (T_2_). The chalkiness area and degree in *OsGAPC3* homozygous mutants increased, but the chalkiness rate did not (**Figure 4A, B**). Scanning electron microscopy (SEM) of the chalky endosperm of *OsGAPC3* homozygous mutants revealed starch granules to be loosely arranged in small spherical shapes, whereas those in wild-type endosperm were more densely arranged in a polygonal structure (**Figure 4C**). The *OsGAPC3* mutation affects the arrangement of starch granules in the endosperm, increasing its chalky area, adversely affecting appearance quality. Additionally, because of the significant impact of *OsGAPC3* mutation on rice yield per plant, we tested the seeds of *OsGAPC3* mutants; their grain length, width, and thickness showed no significant changes (**Supplementary Figure S5**).

To investigate if *OsGAPC3* affects grain cooking and eating quality, the amylose content (AC), gel consistency (GC), taste value, and gelatinization temperature (GT) of *OsGAPC3* mutants (T_2_) was examined. Compared with wild type, the gel consistency and taste value of *OsGAPC3* homozygous mutants reduced significantly (**Figures 4D, E**), but the gelatinization temperature and amylose content did not (**Figures 4F, G**). There was no change in milling quality traits of *OsGAPC3* mutants (**Supplementary Figure S6**). These results indicate that the *OsGAPC3* mutation significantly reduces the gel consistency and taste value of rice, thereby reducing cooking and eating quality.

### 3.6 Proteomic analysis of *OsGAPC3* mutant endosperms

**Figure 5:**
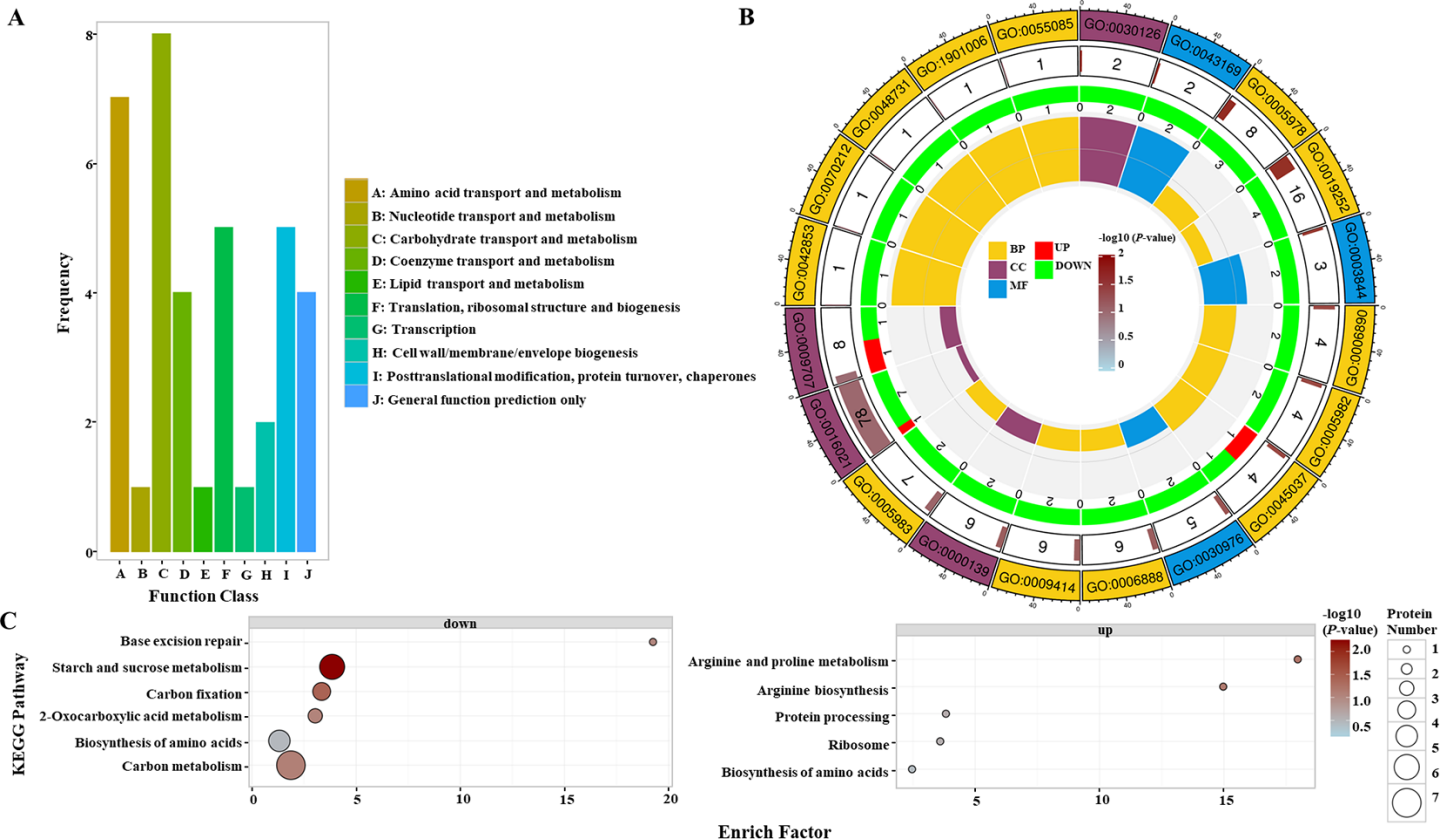
Proteomic analysis of the endosperm of *OsGAPC3* mutants. **(A)** COG functional annotation analysis of differentially expressed proteins (DEPs), with the horizontal axis representing the homologous protein cluster of COG, and the vertical axis representing the number of DEPs annotated to that cluster. **(B)** Differential expression protein GO functional enrichment analysis; the first circle represents the GO number, the second the number of proteins enriched in the GO entry among all proteins, the second circle represents the significance of differential protein enrichment in the GO entry, the darker the color, the higher the significance; the third represents the number of differential proteins enriched in the GO entry, where red represents upregulated proteins, green represents downregulated proteins; and the fourth represents the proportion of differential proteins enriched in the GO entry to all proteins. **(C)** Up- and downregulated DEPs in KEGG pathways, where each circle represents a KEGG pathway, the vertical axis represents the pathway name, and the horizontal axis represents the enrichment factor.

The grain protein content of *OsGAPC3* homozygous mutants was increased. To clarify the composition of and changes in proteins in the endosperm of *OsGAPC3* mutants, we performed proteomic detection and analysis. Proteome detection data are stable, with low variability, and good repeatability within groups (**Supplementary Figure S7A**). The significance of any difference in proteins in samples was judged by the Fold Change (FC) of the repeated quantification value and the *P*-value corresponding to the relative quantification value. Proteins with FC ≥ 1.50 and *P*-value ≤ 0.05 were up-regulated, while those with FC ≤ 1/1.50 and *P*-value ≤ 0.05 were down-regulated. A total of 63 differentially expressed proteins (DEPs) were screened in the endosperm of *OsGAPC3* mutants (**Supplementary Figure S7B**, **Supplementary Table S5**). Most of these 63 DEPs occurred in the nucleus, cell membrane, and cytoplasm (**Supplementary Figure S7C**). These DEPs were functionally annotated in the COG database; most were involved in amino acid and carbohydrate transport metabolisms (**Figure 5A**), consistent with results of *OsGAPC3* in regulating the expression of main storage substances (starch and protein) metabolism-related genes in the early stage (**Supplementary Figure S4**). DEPs were annotated and enriched by Gene Ontology (GO); most of those in the endosperm of *OsGAPC3* homozygous mutants constituted cells, cellular components, and organelles, with molecular functions such as catalytic activity in metabolic, cellular and single-organism processes (**Figure 5B**). To determine the main biochemical metabolic pathways involved in these 63 DEPs, Kyoto Encyclopedia of Genes and Genomes (KEGG) annotation were performed. DEPs downregulated in the endosperm of the mutants of *OsGAPC3* were mainly involved in metabolic pathways such as starch and sucrose metabolism, carbon fixation in photosynthetic organisms, and carbon metabolism, while those that were up-regulated in the endosperm were mainly involved in the metabolic pathways of protein processing, ribosomal pathway, and amino acid biosynthesis in the endoplasmic reticulum (**Figure 5C**). These results show that the DEPs in the endosperm of *OsGAPC3* mutants were significantly downregulated in starch anabolism-related metabolic pathways and upregulated in protein processing and synthesis-related metabolic pathways—a result that is consistent with the phenotype of a significant decrease in total starch content and a substantial increase in grain protein content of the *OsGAPC3* homozygous mutants. According to functionally characterized rice genes (https://venyao.xyz/funRice Genes/), we further analyzed the genes corresponding to these 63 DEPs. Genes such as *OsSUS3*, *OsAlaAT1*, *FLO6*, *SSIIa*, *OsSBEIIb*, *FLO13*, *SBE1*, and *OsARG* are involved in regulating protein and starch metabolism in seeds (**Supplementary Table S6**). Therefore, *OsGAPC3* participates in biological processes such as the synthesis and accumulation of main storage substances (starch and protein) in rice endosperm.

### 3.7 *OsGAPC3* mutation leads to a decrease in rice salt tolerance

**Figure 6:**
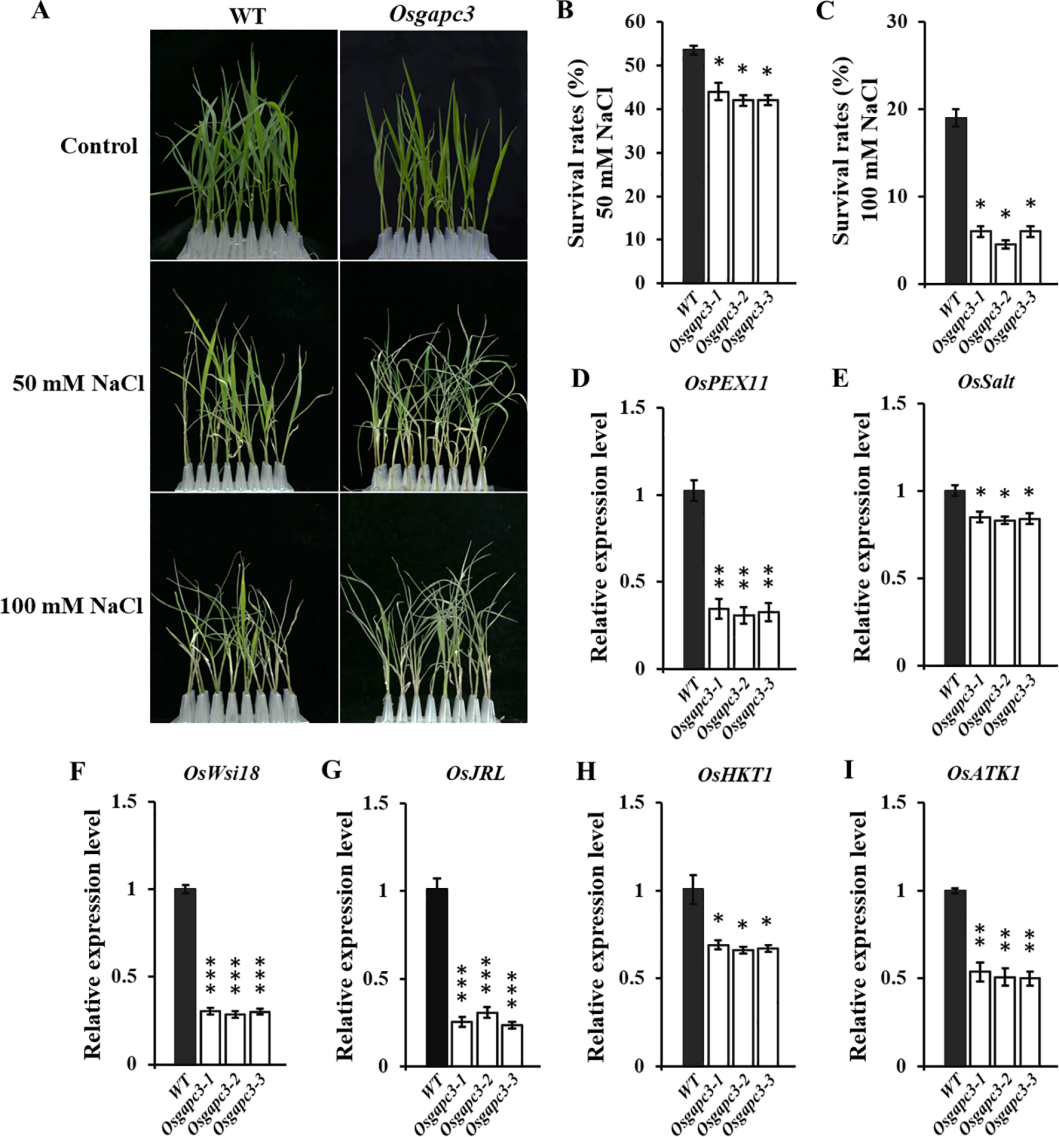
Salt-stress experiments on *OsGAPC3* mutants. **(A)** Salt-stress test. Statistics on the survival rate of rice seedlings at **(B)** 50 mmol·L^−1^ NaCl and **(C)** 100 mmol·L^−1^ NaCl. **(D–I)** qPCR analysis of salt-stress-related genes. Significant differences based on two tailed *t*-test: ^***^ *P* ≤ 0.001, ^**^ *P* ≤ 0.01, ^*^ *P* ≤ 0.05. WT, Wild type. Error bars, standard error of the mean.

As an important enzyme in the biological glycolysis pathway, GAPC3 may be involved in the salt-stress resistance-response. Therefore, we conducted salt-stress experiments on *OsGAPC3* mutant seedlings. Rice seedlings that had grown normally for 14 days were treated with NaCl for 7 d. Survival rates of *OsGAPC3* homozygous mutant seedlings were markedly reduced compared to wild type Zhonghua 11 (**Figures 6A–C**), indicating that the *OsGAPC3* mutation reduced rice-seedling salt-stress resistance. To reveal the molecular mechanism of *OsGAPC3* in the process of salt-stress resistance, we detected expression levels of six genes that related to salt stress in different plant lines (**Supplementary Table S7**). qPCR results indicate that expression levels of genes that positively regulate salt stress were significantly reduced in *OsGAPC3* homozygous mutants (**Figures 6D–J**), which is consistent with the significantly reduced salt-stress resistance of the *OsGAPC3* homozygous mutant.

## 4 Discussion

In the family of plant *AATs*, *AAPs* are most extensively studied. AAPs are involved in lots of physiological metabolic processes, and affect the growth, development, and material metabolism of rice (Fang et al., 2021). In total, 19 *AAPs* have been identified, a majority of which have been cloned in rice (Taylor et al., 2015; Lu et al., 2018; Wang et al., 2019; Ji et al., 2020; Wang et al., 2020; Fang et al., 2021; Wei et al., 2021; Gomes et al., 2022; Yang et al., 2022; Nayak et al., 2023; Yang et al., 2023, 2023). Among them, the *OsAAP6* is a major QTL gene in rice that we previously isolated and cloned to positively regulate the GPC and grain nutritional quality (Peng et al., 2014). Through yeast one-hybrid experiments, 92 regulatory factors that may interact with the *OsAAP6* promoter were screened (Peng et al., 2023). Subsequently, the interaction between *OsGAPC3* and *OsAAP6* was further validated via local surface plasmon resonance experiments and yeast point-to-point rotation (**Figure 1**), and determined to be involved in regulating the expression of *OsAAP6* gene. Our results provide important clues for a comprehensive understanding of the molecular mechanism of *OsAAP6* gene, as well as valuable genetic resources for molecular breeding based upon it.

Rice is an important source of protein for humans, especially in rice in which amino acids are relatively balanced and easily digested and absorbed (Peng et al., 2017). GPC is a crucial factor determining the grain nutritional quality, and increasing it is important to improve its nutritional quality (Peng et al., 2014; Chen et al., 2018). In plants, GAPC is involved in metabolic regulation; for example, strawberry *FaGAPC2* is involved in regulating biological processes such as fruit ripening (Luo et al., 2020). We report *OsAAP6* to be significantly upregulated in homozygous *OsGAPC3* mutants; a significant increase in GPC and a marked decrease in starch content (**Figure 3**) indicates that *OsGAPC3* affects the accumulation of major storage substances (protein and starch) in rice grains. A decrease in the number of PB I, and an increase in the area and quantity of PB II in the rice endosperm of the *OsGAPC3* mutants (**Figure 3**) also suggests that the increase in GPC in *OsGAPC3* mutants is because of the accumulation and increase in volume of PB II in its endosperm. Proteomic sequencing of *OsGAPC3* mutants and detection of protein and starch metabolism-related gene-expression levels revealed *OsGAPC3* to be involved in regulating the expression of protein- and starch-synthesis metabolism-related genes, thereby affecting the accumulation of its main storage substances (starch and protein) (**Supplementary Figure S8**).

Protein and starch are the main components of rice grains, with starch accounting for approximately 80%–90% of a grain’s dry weight. The starch in rice grains is composed of amylopectin and amylose—the composition and proportion of which greatly affects the grain quality traits of rice (Zhao et al., 2022). The amylose is synthesized by the *Waxy* gene encoding GBSSI in rice endosperm (Zhang et al., 2019), and the amylose content plays a key role in cooking and eating quality (Zhang W. et al., 2022; Pang et al., 2016; Peng et al., 2023). Inhibition of the *SSIIa* gene leads to an increase in grain chalkiness, affecting the grain appearance quality (Zhao et al., 2022; Zhang G. Y. et al., 2011). We report no change in the grain chalkiness rate of *OsGAPC3* homozygous mutants, a notable increase in the chalkiness degree and chalkiness area (**Figure 4**), and a significant decrease in the taste value and gel consistency (**Figure 4**). This suggests that increased GPC and decreased starch content in *OsGAPC3* homozygous mutants are the cause. Therefore, the *OsGAPC3* mutation affects the accumulation of protein and starch in grains, affecting multiple grain qualities such as their appearance, cooking and eating qualities.

GAPC plays a key role in various cells (Shang et al., 2011). In addition to being a critical enzyme in the glycolytic pathway, GAPC can also bind to nucleic acids, proteins, and participate in various biological processes (Zaffagnini et al., 2013). In plants, GAPC is involved in metabolic regulation processes such as pollen and seed maturation, abscisic acid signal transduction, transcriptional regulation, and carbohydrate and amino acid balance regulation (Zheng et al., 2003; Muñoz-Bertomeu et al., 2009). *OsGAPC3* is an upstream regulatory factor of stress response genes *DREB2A*, *Lip9*, and *CatA* in overexpressing plants. Subcellular localization results have also demonstrated *OsGAPC3* to be located in the cytoplasm of onion epidermal cells (Zhang X. H. et al., 2011). After overexpression of *Arabidopsis AtGAPC1* and *AtGAPC2*, the expression levels of heat-induced-related genes were all upregulated under heat-stress conditions, and the heat tolerance of *Arabidopsis* seedlings was significantly enhanced (Kim et al., 2020). *Arabidopsis* GAPC undergoes nuclear translocation under heat induction, binding to and activating a transcription factor (NF-YC10) that regulates heat-induced gene expression in the nucleus, thereby enhancing heat tolerance in *Arabidopsis* (Kim et al., 2022). *TaGAPCp1* can positively regulate the response of wheat to drought stress (Li et al., 2019). We report *OsGAPC3* to not be localized on the cell membrane or within the nucleus of rice (**Figure 2**), however, consistent with Zhang X. H. et al. (2011), it may be localized in the cytoplasm. In the salt-stress experiment, we determined that the resistance of *OsGAPC3* mutant seedlings to salt was significantly reduced (**Figure 6**). Under salt-stress conditions, the expression levels of salt-stress-related genes in *OsGAPC3* mutant seedlings are basically consistent with their corresponding salt-stress phenotypes. Thus, under salt stress conditions, *OsGAPC3* may participate in regulating the expression of genes related to salt stress response, thereby affecting the salt stress resistance of its rice.

## 5 Conclusion

OsGAPC3 was screened and confirmed as an important enzyme that can interact with *OsAAP6* gene through yeast one-hybrid, local surface plasmon resonance experiments, and *in vivo* point-to-point rotation experiments. *OsGAPC3* is a constitutively expressed gene in rice, and it may be located in the cytoplasm. The *OsAAP6* are significantly upregulated in *OsGAPC3* homozygous mutants, causing an increase in GPC and improving nutritional quality. *OsGAPC3* is involved in regulating the expression of genes related to the main storage substances (starch and protein) in rice grains, thereby affecting the accumulation of their main storage substances and affecting multiple quality traits. Additionally, *OsGAPC3* may affect the expression of genes related to salt stress in rice, affecting its salt-resistance ability. Therefore, *OsGAPC3* encodes a key enzyme with multiple functions, participating in the regulation of various quality traits, and salt-stress response processes. These results can contribute to improved molecular-design of new high-quality, and salt-resistant rice varieties.

## Data Availability

The data presented in the study are deposited at https://www.cncb.ac.cn/, accession number OMIX007532.
