# Supplementary material for: The OsGAPC3 mutation significantly affects grain quality traits and improves the nutritional quality of rice
Source: Front Plant Sci. 2024 Oct 3;15:1470316. doi: 10.3389/fpls.2024.1470316 (PMC11484083; doi:10.3389/fpls.2024.1470316)
Supplement: Supplementary file 1 [file DataSheet1.docx]

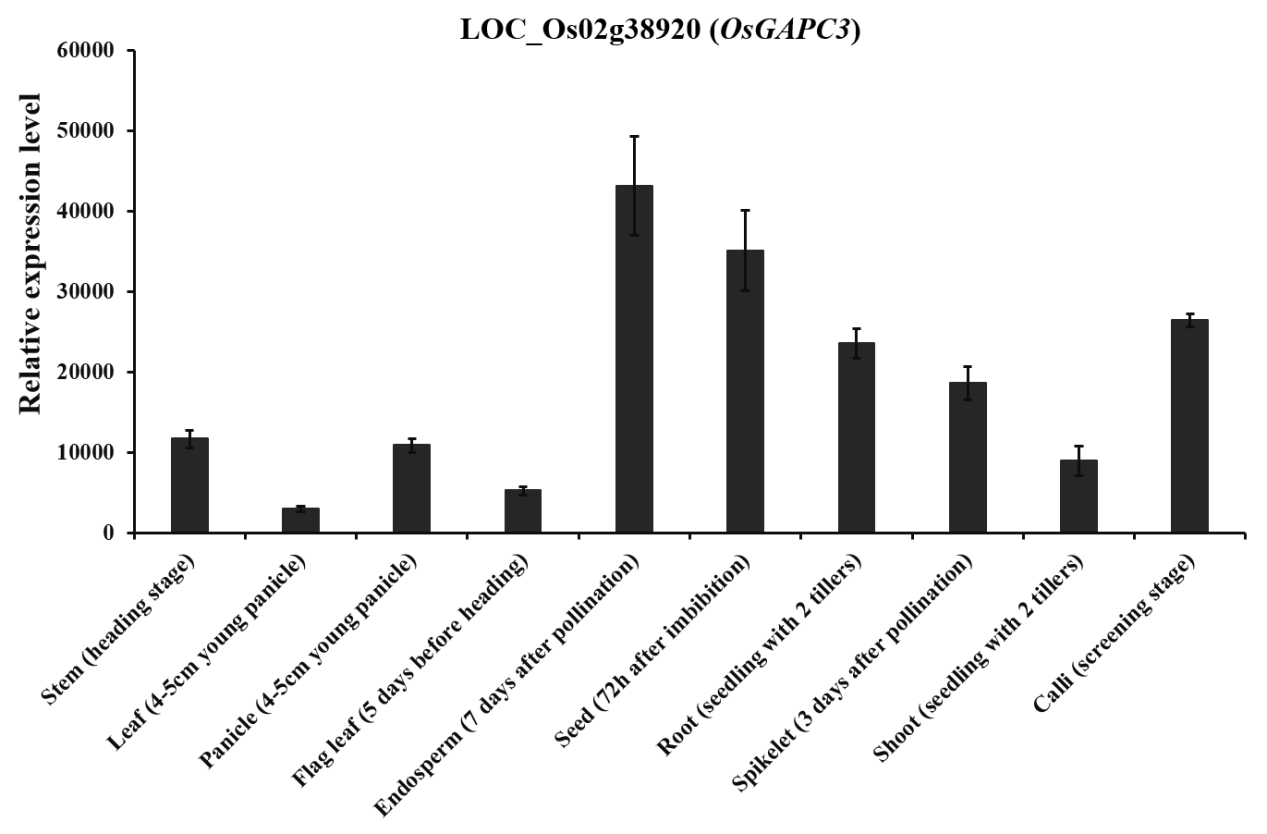


**Figure S1.** The distinct tissue expressions of *OsGAPC3* (LOC_Os02g38920) on CREP (http://crep.ncpgr.cn/crep-cgi/query_by_tree.cgi), and this result is in accordance with Fig 2A.


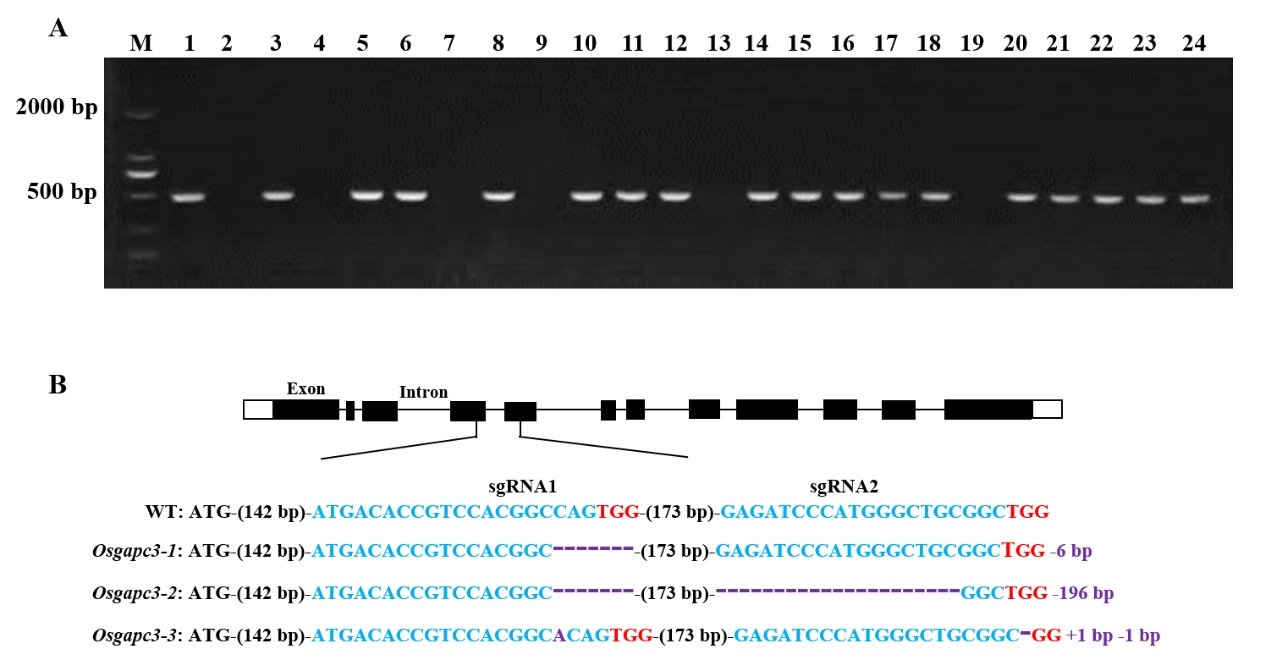


**Figure S2.** Analysis of *OsGAPC3* mutation sites in rice. (A) Positive identification of *OsGAPC3* mutants. Marker: 2000 bp. (B) Sequencing of *OsGAPC3* mutants. Black box, exons; line, introns; white box, non-coding regions. WT: Wild type.


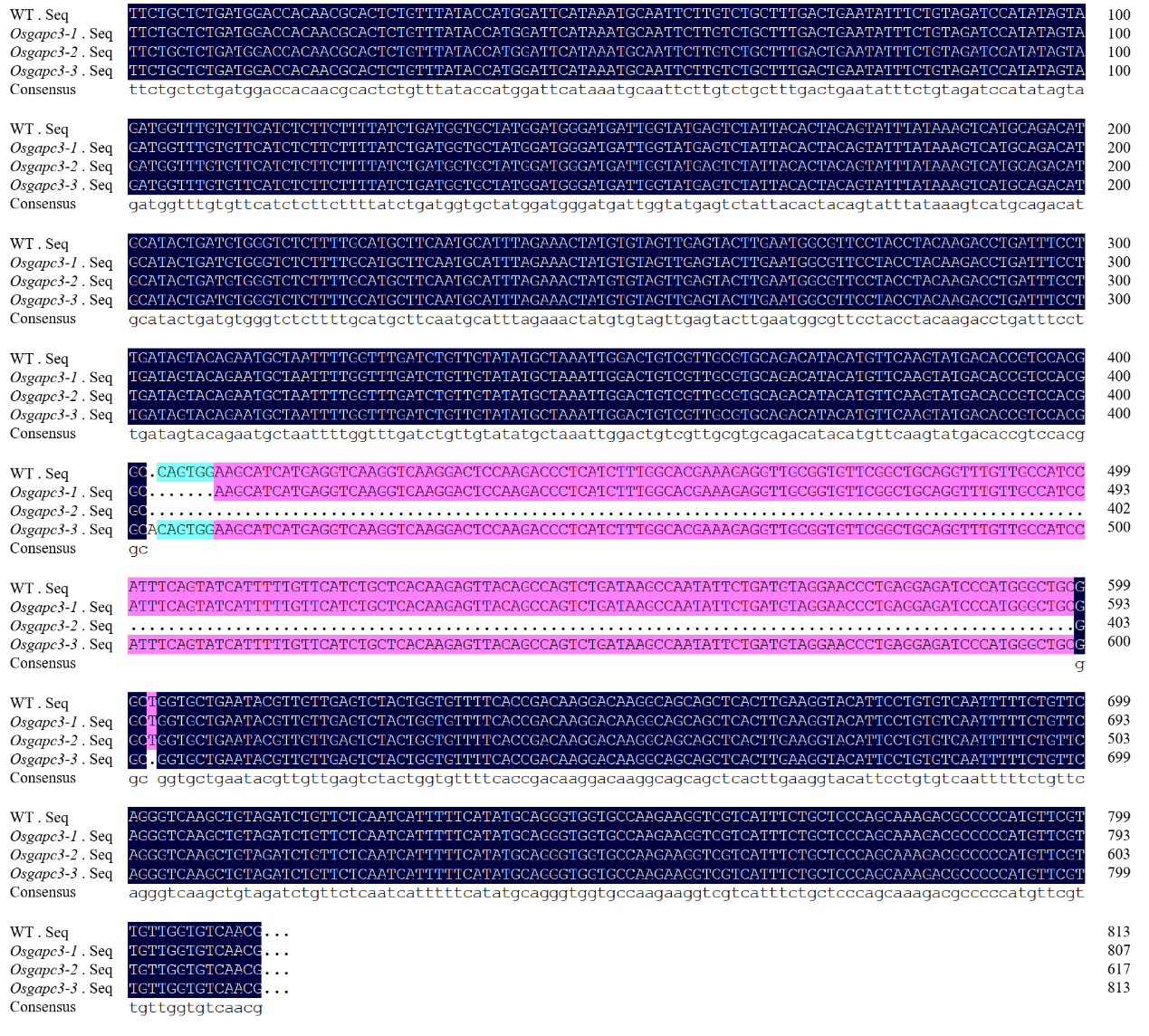


**Figure S3.** Sanger sequencing multi-sequence alignment analysis of *OsGAPC3* mutants. WT: Wild type.


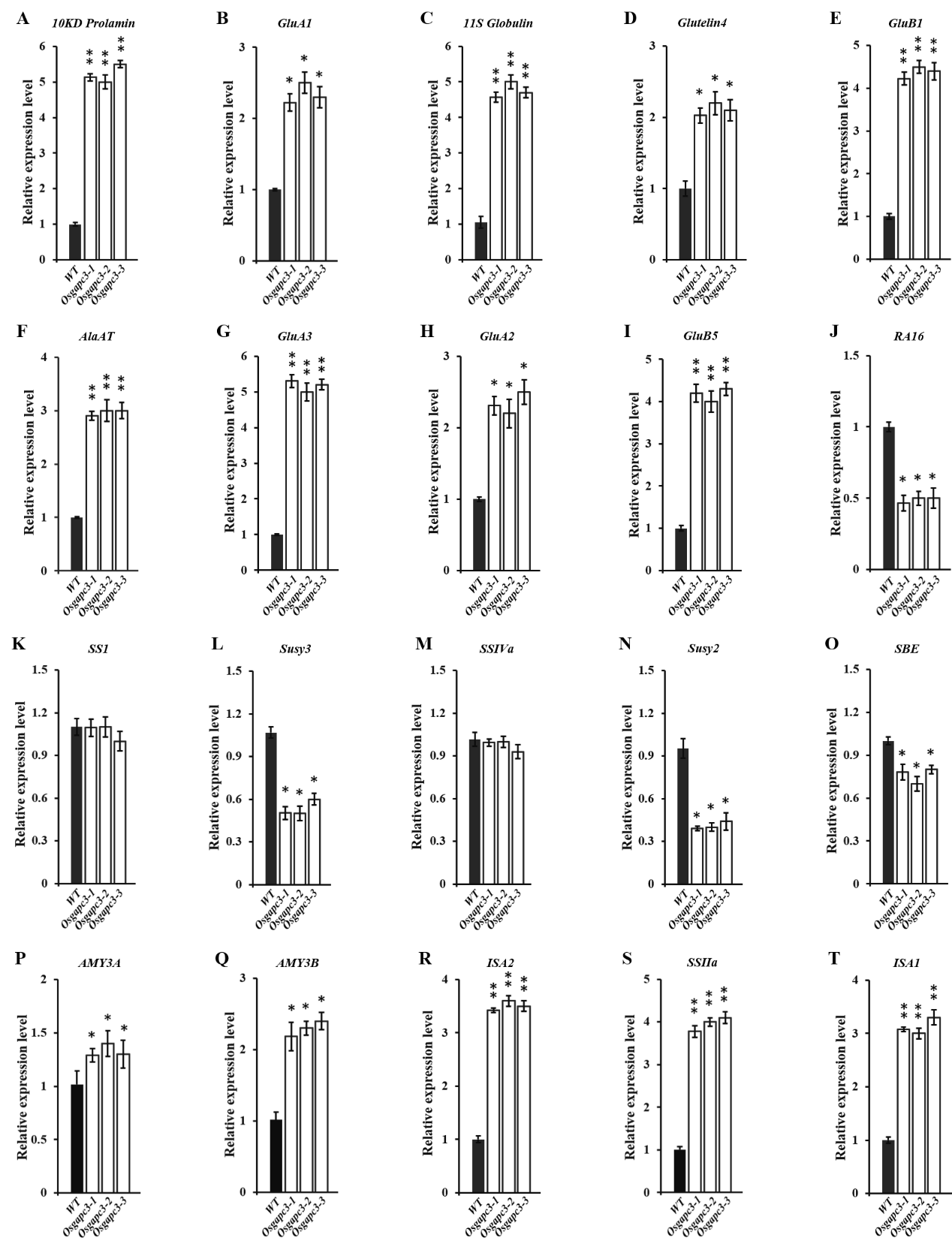


**Figure S4.** Expression analysis of protein and starch metabolism related genes in *OsGAPC3* mutants. (A–J) qPCR analysis of protein-metabolism-related genes. (K–T) qPCR analysis of starch-metabolism-related genes. Significant differences based on two-tailed *t*-test: ^**^*P* ≤ 0.01, ^*^*P* ≤ 0.05. WT: Wild type. Error bars, standard error of the mean.


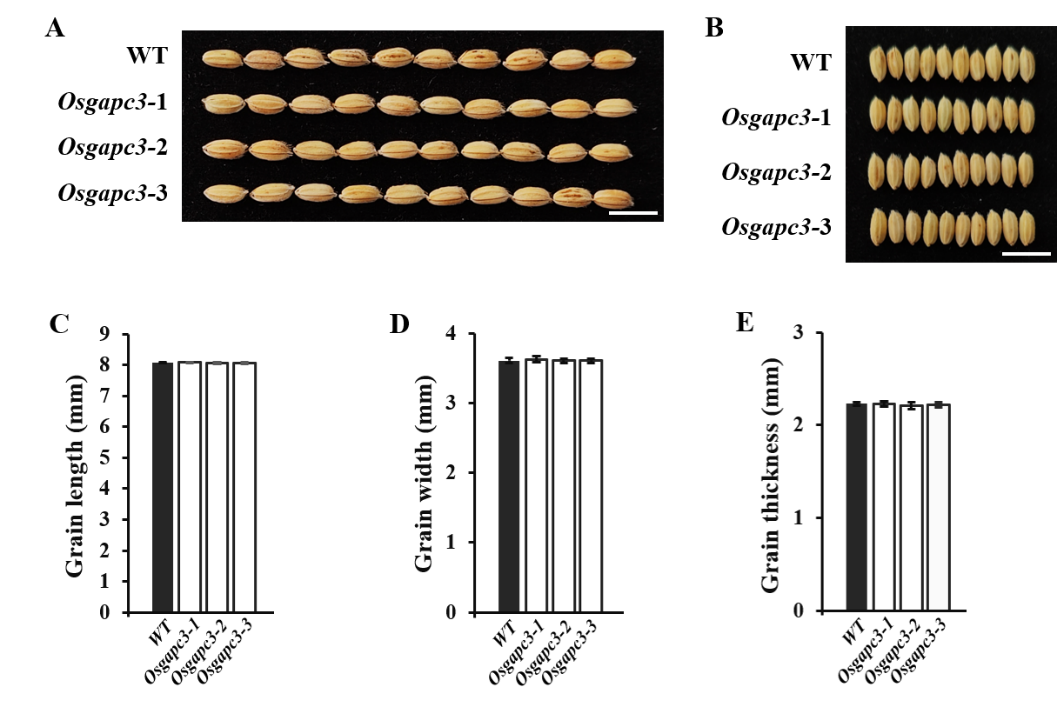


**Figure S5.** Statistical analysis of grain type of *OsGAPC3* mutant seeds. (A) The grain length phenotype of *OsGAPC3* mutant seeds. (B) The grain width phenotype of *OsGAPC3* mutant seeds. (C) Grain length statistics of *OsGAPC3* mutant seeds. (D) Grain width statistics of *OsGAPC3* mutant seeds. (E) Grain thickness statistics of *OsGAPC3* mutant seeds. Bars: 1 cm, Significant differences were based on a two tailed *t*-test. WT: Wild type. Error bars, Standard error of the mean (SEM).


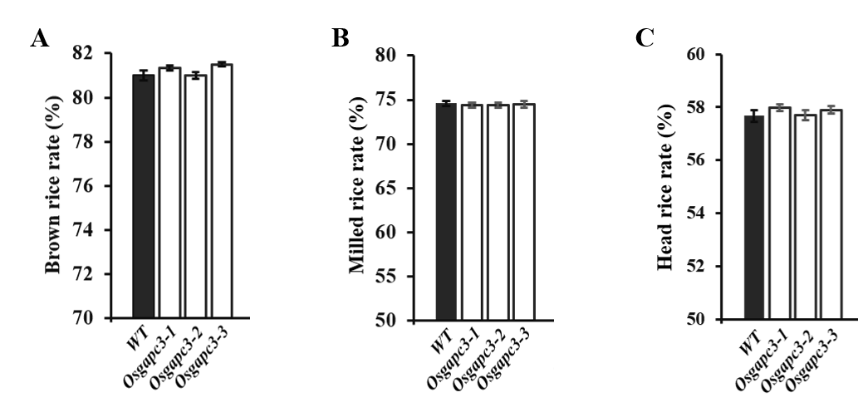


**Figure S6.** Statistical analysis of processing quality traits of *OsGAPC3* mutant rice. (A) Detection and analysis of brown rice percentage in *OsGAPC3* mutant rice. (B) Detection and analysis of polished rice rate in *OsGAPC3* mutant rice. (C) Detection and analysis of whole head rice percentage in *OsGAPC3* mutant rice. Significant differences were based on a two tailed *t*-test. WT: Wild type. Error bars, Standard error of the mean (SEM).


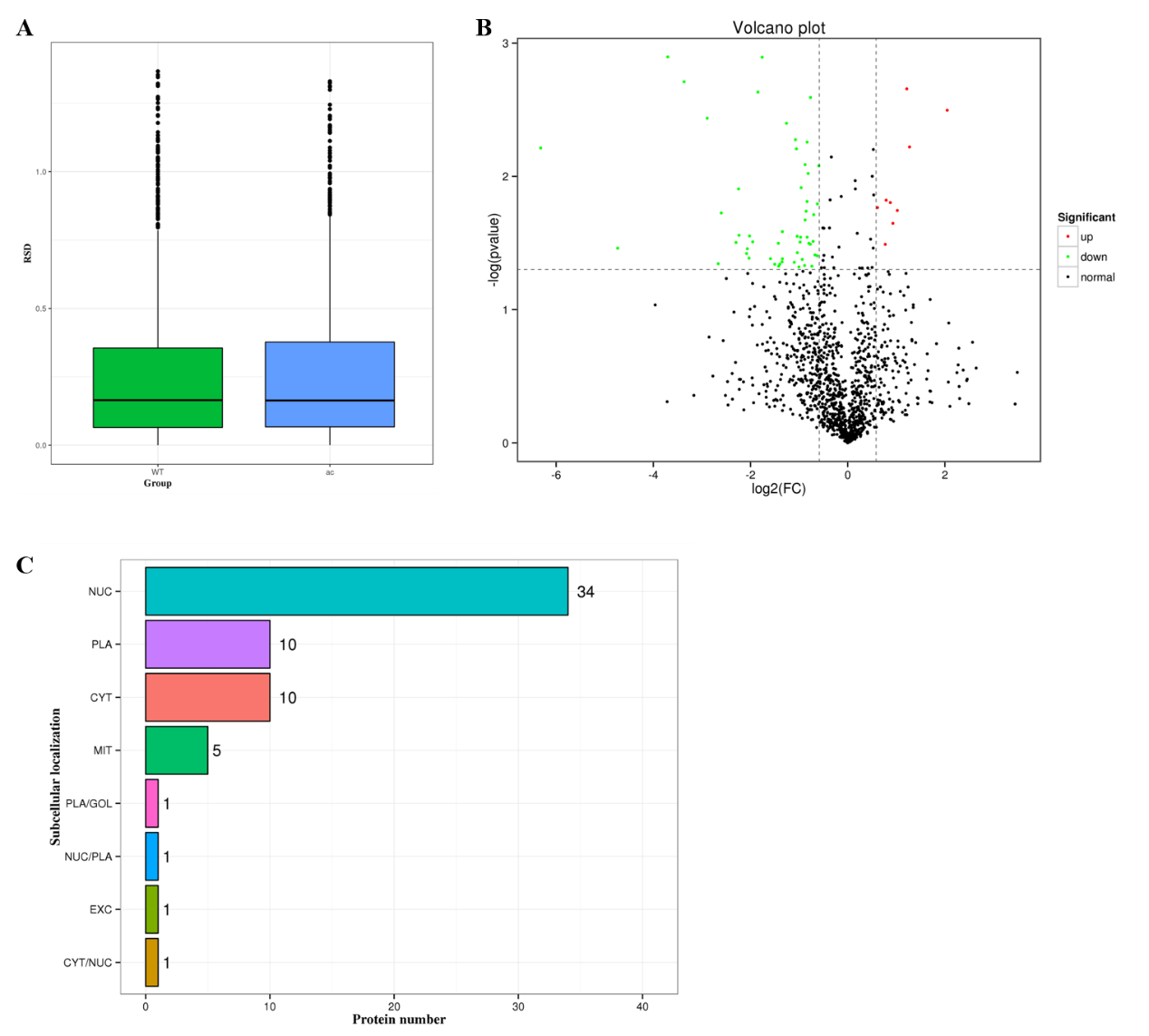


**Figure S7.** Proteomic analysis of the endosperms of *OsGAPC3* mutants. (A) Distribution of relative standard deviation (RSD) for grouping WT and *OsGAPC3* mutants. (B) Volcano plot of DEPs in the endosperm of *OsGAPC3* mutants, with each point representing a gene. (C) Subcellular localization and enrichment analysis of DEPs.


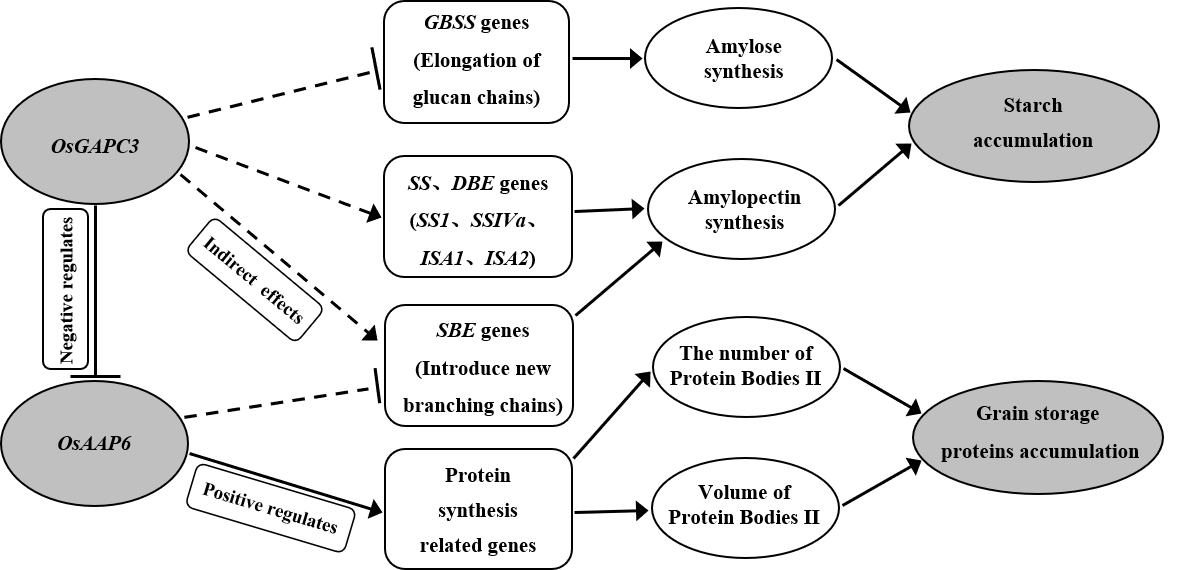


**Figure S8.** Pattern diagram of *OsGAPC3* involvement in regulating protein and starch synthesis and accumulation in rice grains.

Table S1. Primer sequences for mutant creation

| Primer name | Primer sequence（5'-3'） |
| --- | --- |
| sgRNA-F  sgRNA-R  K-F  K-R | ATGACACCGTCCACGGCCAGTGG  GAGATCCCATGGGCTGCGGCTGG  TTCTGCTCTGATGGACCACA  CGTTGACACCAACAACGAAC |

Table S2. Primer sequences used for subcellular localization

| Primer name | Primer sequence（5'-3'） |
| --- | --- |
| *OsGAPC3* | F: TGGGGTACCAATGGCGAAGGAACCGATG  R: CGCTCTAGATTAGTTGAGGCATGAGTAAG |

Table S3. Primer sequences used for protein related genes

| Name | Primer sequence(5'to3') |
| --- | --- |
| *10KD Prolamin-F* | TGCAGTATTTCCCACCAACA |
| *10KD Prolamin-R* | ACATGAACATGGCTGTGGAG |
| *GluB1-F* | GCCAAAGTCAGAGCCAAAAG |
| *GluB1-R* | GAACCAATGTGCAACACCAG |
| *GluA1-F* | CATTTGAGCCAATTCGGAGT |
| *GluA1-R* | GGCCTGATTGTTGGAACTGT |
| *11S Globulin-F* | CACCAAACCCGATCTTCAGT |
| *11S Globulin-R* | CGGAACAGCTTCTCCATCTC |
| *Glutelin4-F* | GTCTAGACGAGATTATGT |
| *Glutelin4-R* | CCTTGTGATCCTTCCAC |
| *AlaAT-F* | CCATTTCTCGAGCAACAACA |
| *AlaAT-R* | ATTTGCAGGGTATCCGTCAC |
| *GluA3-F* | TGAAAACCAACCCTGACTCC |
| *GluA3-R* | ACTCATCTCCCCTCTTGTGC |
| *GluA2-F* | GCAAGAGCAGGAACAAGGAC |
| *GluA2-R* | CCTCATGGTGCAAAAGGTCT |
| *GluB4-F* | GCGACCAGAAGGCTACAAAG |
| *GluB4-R* | TTGCTTGTTGATCGTTGCTC |
| *RA16-F* | AGGTAGTGATCTCGGCGTTG |
| *RA16-R* | CCGATTCCTGGCTGACATAG |
| *β-actin-F* | TGCTATGTACGTCGCCATCCAG |
| *β-actin-R* | AATGAGTAACCACGCTCCGTCA |

Table S4. Primer sequences used for starch related genes

| Gene | Primer sequence(5'to3') |
| --- | --- |
| *SSI-F* | TCATGGATGTGAAGGAGCAA |
| *SSI-R* | TGGCAGTGAACCACAAACAT |
| *Susy3-F* | CATGTACCCCCTGCTCAACT |
| *Susy3-R* | GTCAGCTGTAATGCCTGCAA |
| *SSIVa-F* | GGGAGCGGCTCAAACATAAA |
| *SSIVa-R* | CCGTGCACTGACTGCAAAAT |
| *Susy2-F* | GCTGAAGGACAGGAACAAGC |
| *Susy2-R* | CACCACAGACAACCACAAGG |
| *SBE-F* | GGCATTGCACTCCAAAAGAT |
| *SBE-R* | GCTCCAGTTGTTGCCTTCTC |
| *AMY3A-F* | CCCAGGAGTACCATGCATCT |
| *AMY3A* -*R*  *AMY3B -F* | CTTGGTGATGACCCTCTCGT AGCGGTCTCAGAGTTCCTGCA |
| *AMY3B-R* | TCAAATCTTATTCCAGGCACCA |
| *ISA1-F* | TGCTCAGCTACTCCTCCATCATC |
| *ISA1-R* | AGGACCGCACAACTTCAACATA |
| *ISA2-F* | TAGAGGTCCTCTTGGAGG |
| *ISA2-R* | AATCAGCTTCTGAGTCACCG |
| *SSIIa-F* | GATCGACCAGGATGACGATT |
| *SSIIa-R* | GGGTAAAGCACCTGCAACAT |

Table S5. Differentially expressed proteins in proteomics

|  | Protein ID | log2 FC | *p*-value | Protein regulated |
| --- | --- | --- | --- | --- |
| 1 | Q8H3I3 | -0.628320174 | 0.03945238 | down |
| 2 | Q43009 | -1.055439283 | 0.006216701 | down |
| 3 | Q9LSU1 | -0.700693655 | 0.019399272 | down |
| 4 | Q6AUN1 | -1.345192686 | 0.041551621 | down |
| 5 | C7IZI5 | -1.100373044 | 0.044154141 | down |
| 6 | Q5ZA96 | -0.675099652 | 0.038898094 | down |
| 7 | Q8H920 | -1.760066559 | 0.001276814 | down |
| 8 | Q0DRB3 | -4.73374185 | 0.034608392 | down |
| 9 | A0A0P0XUE4 | -1.345738122 | 0.025983976 | down |
| 10 | A0A0P0W7K6 | -0.76580751 | 0.00255914 | down |
| 11 | Q53N84 | -0.624646683 | 0.016093482 | down |
| 12 | Q67WC9 | -1.848392617 | 0.00233306 | down |
| 13 | Q6Z6L4 | -1.350993424 | 0.043821624 | down |
| 14 | Q0DJ99 | -0.980155402 | 0.031144417 | down |
| 15 | Q7G065 | -0.887460679 | 0.046690419 | down |
| 16 | Q6K2E8 | -1.589621065 | 0.041509121 | down |
| 17 | Q5JLP6 | -0.769503267 | 0.032345819 | down |
| 18 | Q5W740 | 1.271326105 | 0.006017206 | up |
| 19 | Q84MP7 | -2.300022578 | 0.031360542 | down |
| 20 | Q6YZA9 | 0.61199646 | 0.017166453 | up |
| 21 | Q84Q83 | -0.594742795 | 0.008338106 | down |
| 22 | Q7X7N2 | 0.790784065 | 0.015100477 | up |
| 23 | Q6L4X5 | -2.247110875 | 0.012429811 | down |
| 24 | Q5TKJ2 | -0.8366019 | 0.015442844 | down |
| 25 | Q0J0N6 | -0.958488777 | 0.012164961 | down |
| 26 | Q6ZBF6 | -2.082219389 | 0.037955421 | down |
| 27 | Q6Z702 | -0.970747368 | 0.028633833 | down |
| 28 | Q6K5G8 | -6.318412813 | 0.006128695 | down |
| 29 | Q5W6H1 | -1.955283807 | 0.031021816 | down |
| 30 | Q8H903 | -1.426964339 | 0.03180257 | down |
| 31 | Q2QVG9 | -1.420837521 | 0.047217501 | down |
| 32 | A0A5S6R7A0 | -1.396399435 | 0.045569386 | down |
| 33 | Q9AUR8 | -1.037145751 | 0.037381148 | down |
| 34 | Q6Z782 | -0.799974433 | 0.031806134 | down |
| 35 | Q10T66 | 1.215307419 | 0.002205036 | up |
| 36 | Q75KR1 | -1.075427643 | 0.005308757 | down |
| 37 | Q0JMH0 | -0.936485562 | 0.042037832 | down |
| 38 | Q7XUK6 | -2.665636172 | 0.045242756 | down |
| 39 | A0A0P0V9X2 | -3.36735097 | 0.00195107 | down |
| 40 | A0A0P0W399 | -0.835286996 | 0.028601808 | down |
| 41 | Q0E0Q3 | -2.06605487 | 0.034945284 | down |
| 42 | Q5N9F1 | -0.712312576 | 0.030766073 | down |
| 43 | Q53JR9 | -0.835450766 | 0.005537517 | down |
| 44 | Q5QLS8 | -2.236932947 | 0.027692101 | down |
| 45 | Q42456 | -0.73748825 | 0.047205168 | down |
| 46 | Q0DDE3 | -2.030239953 | 0.041134163 | down |
| 47 | Q7XEQ3 | 0.772580703 | 0.032385733 | up |
| 48 | Q69UF2 | -0.877268591 | 0.008167304 | down |
| 49 | A0A0N7KFE7 | -1.259937465 | 0.004001056 | down |
| 50 | B9FM64 | -1.004308311 | 0.047854763 | down |
| 51 | Q5VRY1 | 0.876673252 | 0.015736733 | up |
| 52 | Q6ZFJ0 | 1.020647911 | 0.018044716 | up |
| 53 | Q67W29 | -2.891747146 | 0.00366509 | down |
| 54 | Q6K6A4 | -2.019381539 | 0.028021561 | down |
| 55 | Q65XV6 | 0.929692205 | 0.022539174 | up |
| 56 | A0A0P0VG07 | -0.856402045 | 0.018280835 | down |
| 57 | Q851Y9 | -3.703875557 | 0.001270216 | down |
| 58 | Q67UE5 | -2.601944909 | 0.018829687 | down |
| 59 | Q6ER94 | -1.501957066 | 0.045663813 | down |
| 60 | Q6Z6M4 | -1.040707566 | 0.028152142 | down |
| 61 | A0A0P0WVP5 | -0.876439786 | 0.021256087 | down |
| 62 | Q01401 | -0.814712872 | 0.00952236 | down |
| 63 | Q0E2E9 | 2.046828042 | 0.003191716 | up |

Table S6. Differentially expressed proteins associated with starch and protein metabolism

|  | Protein ID | Gene name | Gene ID | Function | Protein regulated |
| --- | --- | --- | --- | --- | --- |
| 1 | Q43009 | *OsSUS3* | LOC_Os07g42490 | Carbon distribution within the grain filling | down |
| 2 | A0A0P0XUE4 | *OsAlaAT1* | LOC_Os10g25130 | Involved in starch biosynthesis | down |
| 3 | Q7G065 | *FLO6* | LOC_Os01g44220 | Involved in starch biosynthesis | down |
| 4 | Q0DDE3 | *SSIIa* | LOC_Os06g12450 | Involved in endosperm starch synthesis | down |
| 5 | A0A0N7KFE7 | *OsSBEIIb* | LOC_Os02g32660 | Involved in starch biosynthesis | down |
| 6 | Q6K6A4 | *FLO13* | LOC_Os02g57180 | Involved in starch biosynthesis | down |
| 7 | Q01401 | *SBE1* | LOC_Os06g51084 | Involved in starch biosynthesis | down |
| 8 | Q7X7N2 | *OsARG* | LOC_Os04g01590 | Involved in nitrogen transport and storage | up |

Table S7. Quantitative primer of salt stress related genes

| Name | Primer sequence(5'to3') |
| --- | --- |
| *OsPEX11-F* | GCGTCTACTACTTCCTCG |
| *OsPEX11-R* | GACTCCAGTTTGCCGATC |
| *OsJRL-F* | AGGCGTGACAATCTACAG |
| *OsJRL-R* | GGTTCCAGAAATCTCCTTGA |
| *OsHKT1-F* | ACACCCAATATTATTCCTCTTAA |
| *OsHKT1-R* | CGGGAATACGCTAAAGG |
| *OsAKT1-F* | AGAGATCCTTGATTCACTGCC |
| *OsAKT1-R* | TCTACTAACTCCACACTACCAG |
| *Salt-F* | CGAAATAATGTTCCATGGTGTT |
| *Salt-R* | TGTACTACGGATCGGTGCAA |
| *OsWsil8-F* | TGTGACTCGATCCAGCGTAG |
| *OsWsil8-R* | GTTCCTGCTGAGAAGCCATC |
